# Seasonal dynamics of *Amblyomma cajennense* (Fabricius, 1787) sensu stricto in a degraded area of the Amazon biome, with notes on *Rickettsia amblyommatis* infection

**DOI:** 10.1186/s13071-023-05978-9

**Published:** 2023-10-27

**Authors:** Flávio Eduardo Saraiva de Araújo, Thiago Fernandes Martins, Carlos Celso Mendonça Ramos, Rafael Michael Silva Nogueira, João Luiz Horacio Faccini, Mariana Avelar Tavares, Nicolas Jalowitzki de Lima, Eduardo Bezerra de Almeida Júnior, Lucas Christian de Sousa-Paula, Filipe Dantas-Torres, Felipe da Silva Krawczak, Livio Martins Costa-Junior, Marcelo Bahia Labruna, Leonardo Teixeira Dall′Agnol, Hermes Ribeiro Luz

**Affiliations:** 1 Post-Graduation Program in Health and Environment, Federal University of Maranhão, São Luís, MA Brazil; 2 Pasteur Institute, São Paulo State Department of Health, São Paulo, SP Brazil; 3 Department of Preventive Veterinary Medicine and Animal Health, Faculty of Veterinary Medicine and Animal Science, University of São Paulo, São Paulo, Brazil; 4 Post-Graduation Program in Animal Health Defense, State University of Maranhão, Maranhão, Brazil; 5 Post-Graduation Program in Health Sciences, Center of Biological and Health Sciences, Federal University of Maranhão, São Luís, MA Brazil; 6 Veterinary and Animal Science School, Federal University of Goiás, Goiânia, GO Brazil; 7 Post-Graduation Program in Biodiversity and Conservation, Center of Biological and Health Sciences, Federal University of Maranhão, São Luís, MA Brazil; 8 Tick-Pathogen Transmission Unit, Laboratory of Bacteriology, National Institute of Allergy and Infectious Diseases, Hamilton, MT USA; 9 Department of Immunology, Aggeu Magalhães Institute, Oswaldo Cruz Foundation (Fiocruz), Recife, PE Brazil; 10 Post-Graduation Program in Northeast Biotechnology Network (RENORBIO), Biodiversity and Conservation, Center of Biological and Health Sciences, Federal University of Maranhão, São Luís, MA Brazil

**Keywords:** *Amblyomma cajennense*, Ixodidae, Population dynamics, *Rickettsia*, Amazon region, Brazil

## Abstract

**Background:**

The tick *Amblyomma cajennense* sensu stricto (*A. cajennense* s.s.) frequently parasitizes animals and humans in the Amazon biome, in addition to being a vector of *Rickettsia amblyommatis*. In the present study, we evaluated both the population dynamics of *A. cajennense* s.s. in a degraded area of the Amazon biome and the presence of rickettsial organisms in this tick population.

**Methods:**

The study was carried out in a rural area of the Santa Inês municipality (altitude: 24 m a.s.l.), Maranhão state, Brazil. Ticks were collected from the environment for 24 consecutive months, from June 2021 to May 2023. The region is characterized by two warm seasons: a rainy season (November–May) and a dry season (June–October). We characterized the temporal activity of *A. cajennense* s.s. on the vegetation by examining questing activity for each life stage (larvae, nymphs, adults [males and females]) in relation to the dry and rainy season. Ticks collected in this study were randomly selected and individually tested by a TaqMan real-time PCR assay that targeted a 147-bp fragment of the rickettsial *gltA* gene.

**Results:**

Overall, 1843 (62.4%) adults (52.6% females, 47.4% males), 1110 (37.6%) nymphs and 398 larval clusters were collected. All adult females and nymphs were morphologically identified as *A. cajennense* s.s. Larval activity was observed from April to December, with a peak from June to September (dry season); nymph abundance peaked from September to November (transition period between dry and rainy seasons); and adult ticks were abundant from October to May (spring/summer/early autumn). The infection rate by *R. amblyommatis* in *A. cajennense* s.s. ticks was at least 7% (7/99).

**Conclusion:**

Our data suggest a 1-year generation pattern for *A. cajennense* s.s., with a well-defined seasonality of larvae, nymphs and adults in the Amazon biome. Larvae predominate during the dry season, nymphs are most abundant in the dry-rainy season transition and adults are most abundant in the rainy season. The presence of *R. amblyommatis* in adult ticks suggests that animals and humans in the study region are at risk of infection by this species belonging to the spotted fever group of* Rickettsia*.

**Graphical Abstract:**

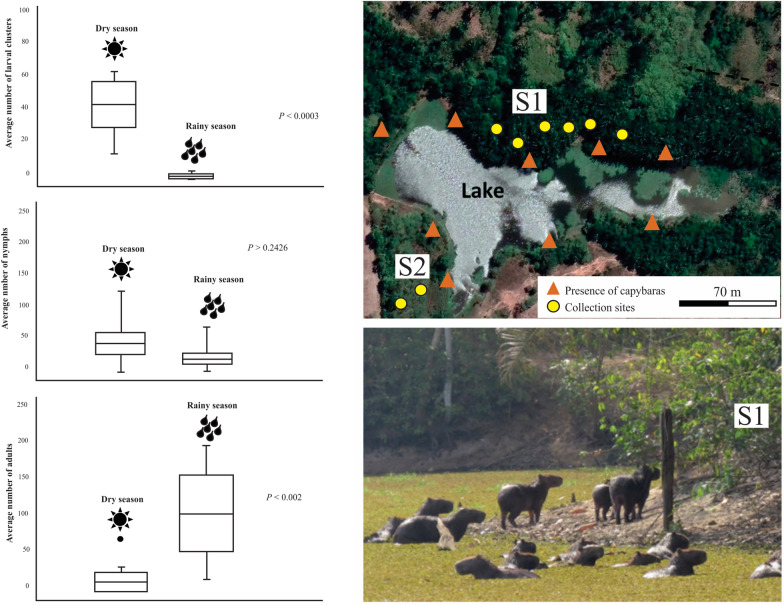

## Background

The transmission dynamics of tick-borne diseases, such as Lyme disease and spotted fevers, involves complex eco-epidemiological interactions between their causative pathogens, tick vectors, the environment and susceptible host populations [1–4]. The abundance and seasonal distribution of ticks (larvae, nymphs and adults) play a major role in the epidemiology of tick-borne diseases [1, 5–7]. Therefore, studies that evaluate these variables contribute to the mitigation of possible factors that could increase the risk of such diseases to animals and humans [8]. For example, the incidence of Lyme disease is strongly linked to the seasonal pattern of its tick vectors [9–11]. Similarly, the incidence of Brazilian spotted fever (BSF) caused by *Rickettsia rickettsii* depends mainly on the phenology of its vector *Amblyomma sculptum* [1, 2].

Abiotic factors, such as temperature, precipitation and humidity, are essential for the survival of ticks during their non-parasitic phase [12–15]. Outside the optimal range of these factors, tick activities (e.g. host-seeking behavior) can be inhibited and, in some cases, lead to tick mortality [6, 16]. Consequently, abiotic factors directly govern the spatial and temporal distribution patterns of ticks [6, 17, 18], as has been shown for *A. sculptum*, for example, whose developmental stages exhibit a pronounced correlation with seasonal fluctuations. Adults are more abundant during the spring and summer (rainfall and high temperatures), larvae during mid-autumn and early winter (less rainfall and low temperatures) and nymphs are more abundant during the winter and early spring (less rainfall and moderate temperatures) [19]. It has been demonstrated that the 1-year generation pattern of *A. sculptum* in southeastern Brazil is controlled by the behavioral diapause of larvae hatching during the spring and summer seasons [20]. These larvae typically start their host-seeking activity in the subsequent autumn [20, 21].

While the population dynamics of *Amblyomma* ticks has been extensively investigated in southeastern Brazil, only a few such studies have been conducted in other regions, such as the Amazon biome where only a single study has evaluated the seasonality of *Amblyomma* ticks [22]. This is a significant research gap, considering that some of the tick species found in this biome are important from a medico-veterinary perspective. This is the case for *Amblyomma cajennense* sensu stricto (*A. cajennense* s.s.), whose distribution is restricted to the Amazon biome [23, 24]. Larvae, nymphs and adults of *A. cajennense* s.s. preferentially feed on large mammals (e.g. *Tapirus terrestris* [South American tapir], *Hydrochoerus hydrochaeris* [capybara], *Tayassu pecari* [white-lipped peccary], *Pecari tajacu* [collared peccary]), which are important hosts for the maintenance and dispersion of these ixodids in nature. Larvae and nymphs are also commonly reported on species of order Carnivora, medium-sized rodents and marsupials, as well as large ground-inhabiting birds. *Amblyomma cajennense* s.s. is an opportunistic tick-borne microbe that can parasitize humans in the Amazon biome [25–27]. Moreover, *A. cajennense* s.s. ticks are often infected (20–26%) by *Rickettsia amblyommatis* (formerly ‘*Candidatus Rickettsia amblyommii*’), which is successfully maintained by transovarial and transstadial transmission [28]. While there is little information on *R. amblyommatis* infections in animals and humans, its role as a causative agent of spotted fever in humans cannot be ruled out [29].

Thus, knowledge of the population dynamics of *A. cajennense* s.s. is fundamental for future acarological and health surveillance measures in the Amazon biome. To fill this gap, we have evaluated the population dynamics of *A. cajennense* s.s. in a degraded area of the Amazon biome as well as the presence of rickettsial organisms in this tick population.

## Methods

### Study area

**Fig. 1:**
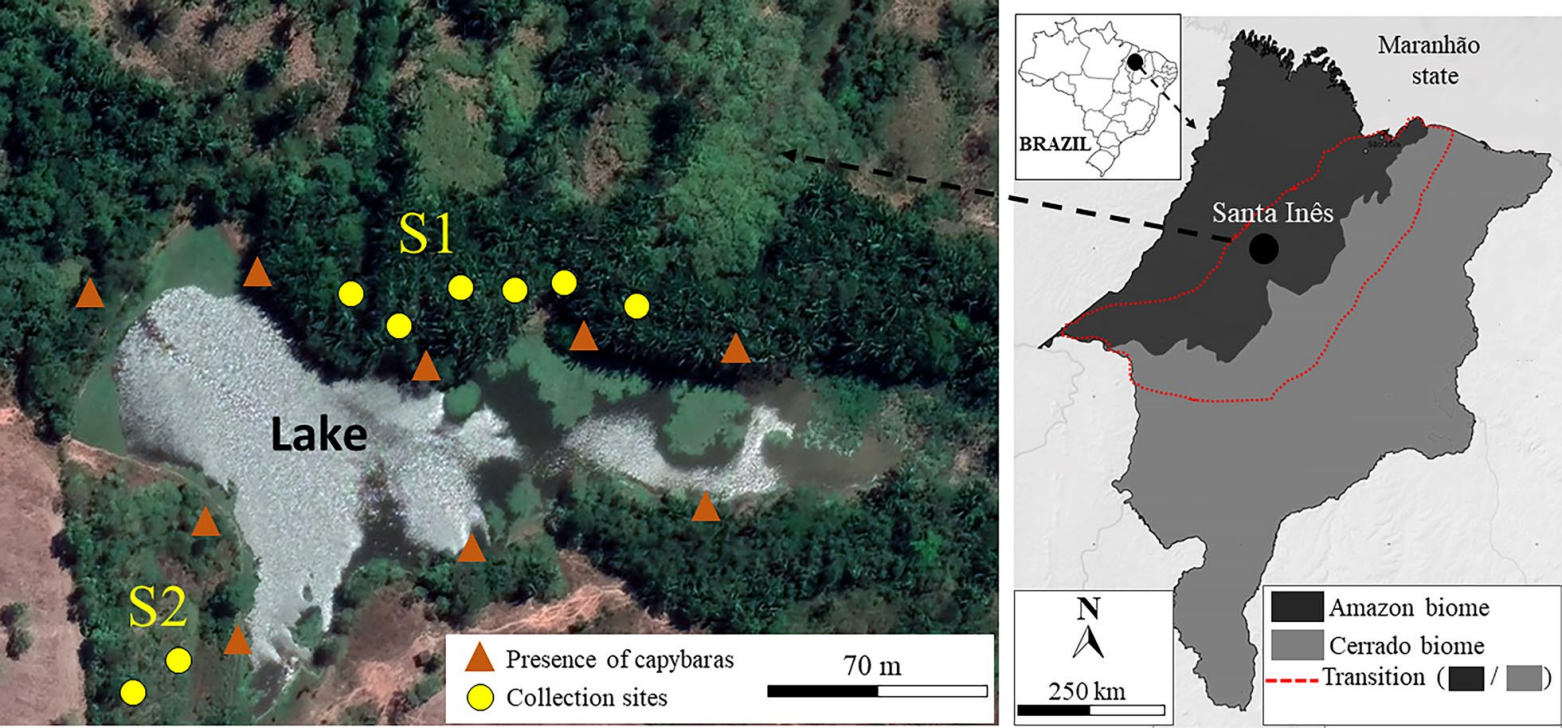
Map showing the study area, municipality of Santa Inês, Maranhão state, Brazil. Transition area was defined following Marques et al. [54] The Eastern Amazon-Cerrado transition area varied from approx. 40 to 250 km. S1, S2, Study sites 1, 2, respectively

**Fig. 2:**
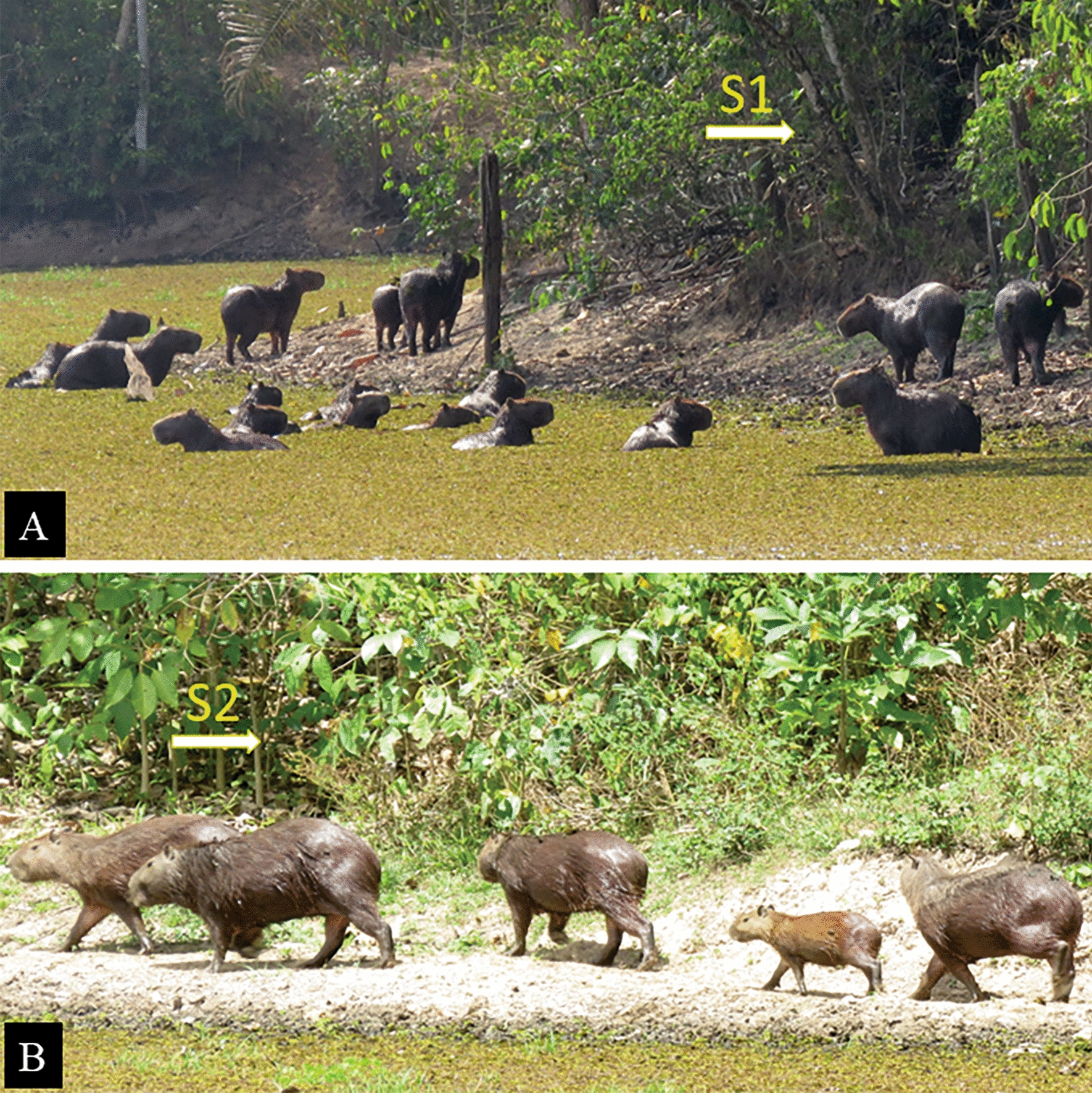
Group of capybaras (*Hydrochoerus hydrochaeris*) in the study area.** A** Adults and juveniles, **B** adults, juveniles and cubs

The study was carried out in a rural area of Santa Inês municipality (altitude: 24 m a.s.l.), Maranhão state. Santa Inês is located in the Amazon biome, and its climate is hot and humid, with an average annual temperature of 27 °C and annual average precipitation of 1710 mm [30]. The region is characterized by two warm seasons: a rainy season from November to May, and a dry season from June to October. The study was carried out at two sites located within a forest fragment, totaling approximately 10,000 m^2^ (Fig. 1). Study site 1 (S1; 03°05′44″S, 45°32′50″W) was characterized as a more closed vegetation area due to the agglomeration of trees, shrubs and lianas. Shrubs and trees (approx. 3–6 m high) included many medium-sized plants, such as fever tree (*Vismia guianensis*), purple jurubeba (*Solanum paludosum*), blackrodwood (*Eugenia biflora*) and young individuals of *Inga* sp., *Pouteria* sp., *Tocoyena* sp., as well as a large amount of ipês (family Bignoniaceae) in the understory; in addition, two species of the large flowering family Fabaceae (*Schnella* sp. and *Bauhinia* sp.) have been recorded. In terms of undergrowth (vegetation approx. 50–100 cm in height), *Cyperus* sp., *Ipomoea* sp., *Alternathera* sp., and whitehead broom (*Borreria verticillata*) were observed. The babassu palm (*Attalea speciosa*) was a dominant plant in the area. Study site 2 (S2; 03°52′48″S, 45°32′53″W) was composed of more open forest and dense herbaceous and undergrowth vegetation, with the presence of Siam weed (*Chromolaena odorata*), blue snakeweed (*Stachytarpheta cayennensis*), Touch-me-not (*Mimosa pudica*), false mallow (*Sida spinosa*) and yellow alder (*Turnera ulmifolia*). An agglomeration of subshrubs and thin-stemmed shrubs was also observed, such as common lantana (*Lantana camara*), spider flower (*Cleome spinosa*), Caesar weed (*Urena lobata*), along with isolated and spaced arboreal elements throughout the area, represented by candle bush (*Senna alata*), gumtree (*Sapium glandulosum*), *Coccoloba* sp. and the cultivated species brown salwood (*Acacia mangium*); in addition, some young individuals of ipês were recorded scattered throughout the area. Between the collection sites there was a lake of around 15,000 m^2^, where it was possible to see groups of capybaras in groups of to 60 individuals (cubs, juveniles and adults) (Fig. 2). Other mammals were also seen in the study area: paca (*Cuniculus paca*), opossum (*Didelphis* sp.), coati (*Nasua nasua*) and agouti (*Dasyprocta* sp.). Humans frequently visited tick-infested areas, so parasitism by ticks was common. *Amblyomma cajennense* s.s. is known to be present in the study area, whereas *A. sculptum* has not yet been recorded [24].

### Tick collection

**Fig. 3:**
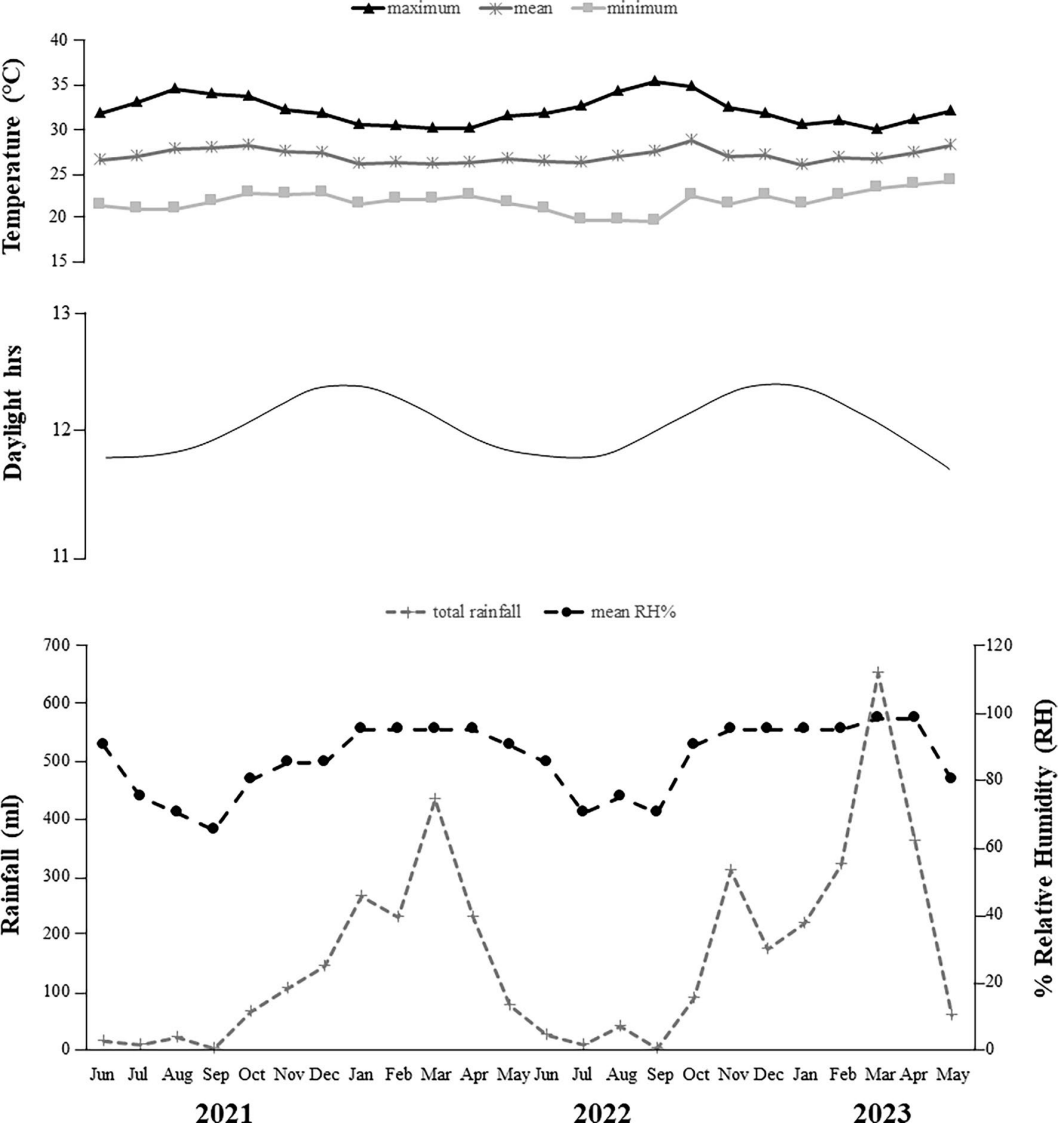
Meteorological data for the study region during the study period (June 2021 to May 2023) were provided by Agritempo/EMBRAPA

Ticks were collected monthly from June 2021 to May 2023, with a total of 24 collections across both the rainy (November–May) and dry seasons (June–October) (Fig. 3). Each month, ticks were collected by visual search and by dragging, as previously described [31]. Collections were performed between 8:00 am and 12:00 pm and between 2:00 pm and 5:00 pm. The dragging method consisted of passing a cotton flannel (2 m long × 1 m wide) over the leaf litter and vegetation. Dragging was performed for approximately 30–40 min along linear transects of approximately 70–100 m, making stops approximately every 5 m to collect ticks from the cotton flannel and those on the researchers' clothes. The visual search method consisted of visually inspecting the vegetation for the presence of ticks [31–33]. Two researchers were needed to carry out the collections by dragging, and three researchers for the visual search method.

Ticks removed from the researchers’ clothing, cotton flannel (dragging method) and vegetation were stored in 15- or 50-ml test tubes containing 70% ethanol and sent to the Laboratory of Parasite Control of the Federal University of Maranhão for identification. Ticks were morphologically examined under a binocular stereoscope (Carl Zeiss AG, Oberkochen, Germany) at a magnification of between 6.3× and 50×. Adults and nymphs were identified to species level using morphological keys and species descriptions [23, 24, 34, 35]. Voucher specimens were deposited in the tick collection “Coleção Nacional de Carrapatos Danilo Gonçalves Saraiva” of the University of São Paulo (accession number CNC-4656).

### Molecular detection of rickettsiae

Of the 3351 ticks collected in this study, 99 adults (50 males, 49 females) were randomly selected and individually processed for DNA extraction using the guanidine isothiocyanate and phenol/chloroform technique [36].

DNA from ticks were tested by a TaqMan real-time PCR assay (Thermo Fisher Scientific, Waltham, MA, USA) that targeted a 147-bp fragment of the rickettsial *gltA* gene, following the protocol previously described by Soares et al. [37]. The real-time PCR-positive samples were subsequently tested with a conventional PCR assay using primers Rr190.70p and Rr190.602n targeting the 190-kDa outer membrane protein gene (*ompA*) of the spotted fever group (SFG) *Rickettsia* spp., as described previously [38]. The *ompA* PCR products were purified using the Wizard_®_ SV Gel and PCR Clean-Up System (Promega, Madison, WI, USA) and sequenced using the BigDye™ Terminator v3.1 Matrix Standards Kit (Applied Biosystems, Thermo Fisher Scientific). Sequencing reactions were carried in both directions in a 3500xL Genetic Analyzer (Applied Biosystems, Thermo Fisher Scientific) using the same primers as for the PCR analyses. The obtained sequences were assembled and analyzed using Sequencher® v. 5.4.6 software (Gene Codes Corp., Ann Arbor, WI, USA), considering a Phred quality score of ≥ 30. Consensus sequences were subjected to BLASTn analyses (www.ncbi.nlm.nih.gov/blast) to infer the closest similarities available in GenBank.

Tick DNA samples that tested negative to rickettsial DNA by the real-time PCR were also tested by the conventional PCR targeting the tick mitochondrial 16S ribosomal DNA (rDNA) gene [39], with the aim to validate the DNA extraction protocol. If the tick sample yielded no product by this PCR, it was considered that DNA extraction was not successful, and the individual tick was discarded from the study.

### Statistical analyses

We characterized the temporal activity of *A. cajennense* s.s. on vegetation by examining questing activity for each life stage (larvae, nymphs, adults [males and females]) in relation to the dry and rainy season. Meteorological data were obtained from Agritempo, an agrometeorological monitoring system provided by the Brazilian Agricultural Research Corporation (Embrapa), using information recorded from meteorological station number 82900 (68 km distant from the study area). Data on temperature (°C) and rainfall total (mm) are presented as monthly averages. Pearson's correlation coefficient was used to compare the number of ticks at each stage (larvae, nymphs, and adults) with the average monthly temperature, relative humidity (RH) and precipitation (mm), from June to September (dry season) and from November to April (rainy season). Means of ticks according to the most significant months of drought and rainfall were compared using the Kruskal–Wallis H-test. Daylight was calculated by subtracting the hours between sunrise and sunset, following the latitude and altitude of the study area. Normality of data was assessed using the Lilliefors test. The level of significance was set at *P* ≤ 0.05. Statistical analyses were performed using BioEstat software, v5.3 (Mamirauá Institute of Sustainable Development, Tefé, AM, Brazil).

## Results

Overall, 1843 (62.4%) adults (52.6% females, 47.4% males), 1110 (37.6%) nymphs and 398 larval clusters were collected. All adult females and nymphs were morphologically identified as *A. cajennense* s.s. Since no other tick species was found in our study, all males and larvae (morphologically compatible with *A. cajennense* sensu lato) were conveniently designated as *A. cajennense* s.s. While ticks were collected in both sites, significantly more were collected in S1 (93.6% of individual ticks and 82.2% of larval clusters) compared to S2 (Chi-square test, *χ*^2^ = 6.68, *df* = 1, *P* < 0.002). In S1, all tick stages (larvae, nymphs and adults) were highly abundant, regardless of the season. Larvae, nymphs and adults were also collected in S2, but the collected ticks were mainly larvae (47 clusters) and, in lower abundance, adults (*n* = 23) and nymphs (*n* = 89). In S2, larval clusters were found exclusively in the dry season.

**Fig. 4:**
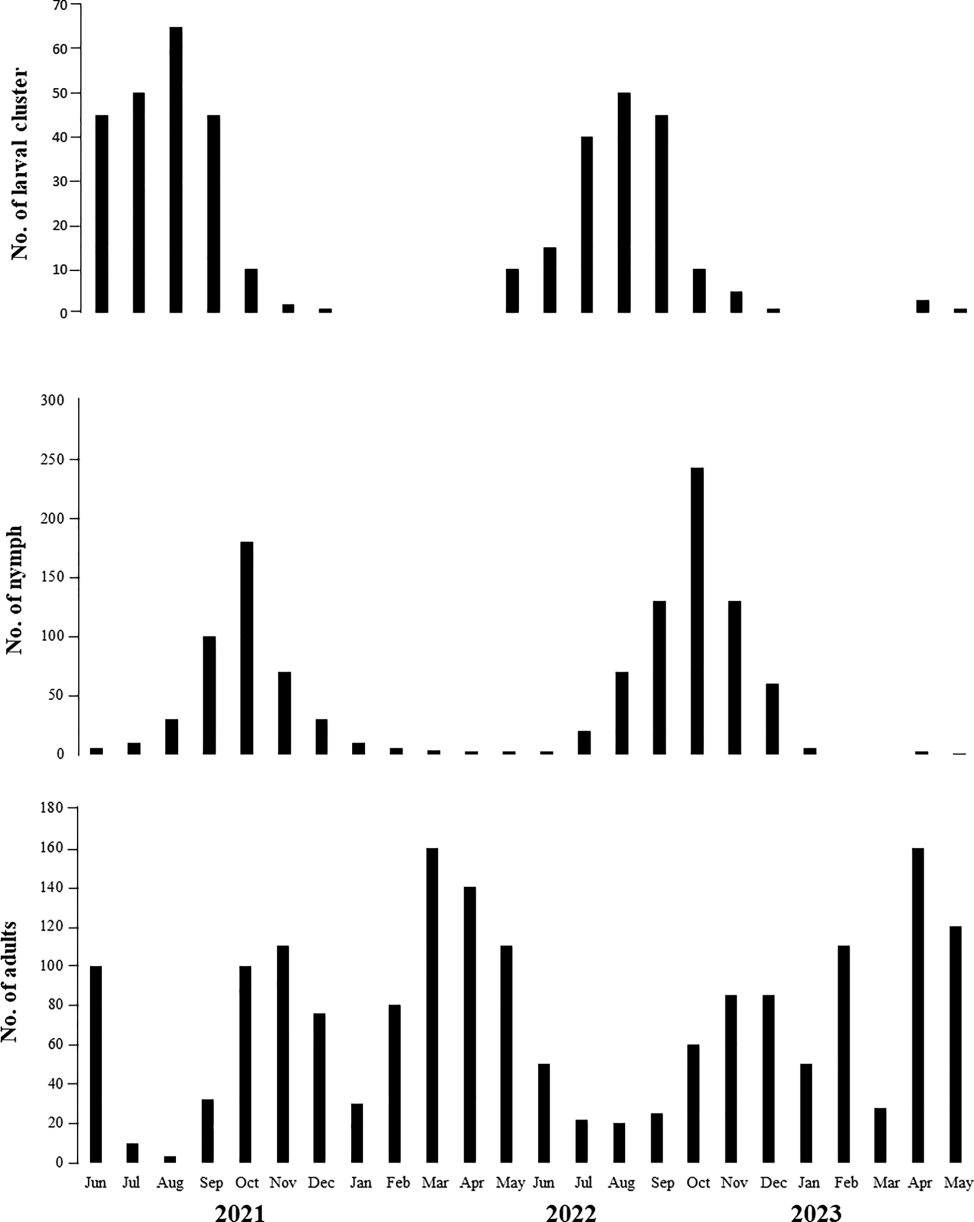
Seasonal activity of larvae, nymphs and adults of *Amblyomma cajennense* sensu stricto in the Amazon biome, municipality of Santa Inês, Maranhão state, Brazil

Adult ticks were collected in all 24 sampled months, whereas nymphs were collected in 22 months (no nymphs collected in February and March 2023) and larvae were collected in 17 months (no larvae collected during the periods January–April 2022 and January–March 2023) (Fig. 4). In both collection years, larval activity was observed from April to December, with a peak from June to September (dry season) (Fig. 4). The highest abundance of larvae in the environment was recorded for August in both study years (65 and 50 larval clusters in 2021 and 2022, respectively). Nymph abundance peaked from September to November (transition period between dry and rainy seasons), with the highest abundance recorded in October of both years (180 and 243 individuals in October 2021 and October 2022, respectively) (Fig. 4). Adult ticks were abundant from October to May (spring/summer/early autumn), with the highest peaks in abundance occurring in March and April of 2022 (156 and 143 individuals, respectively) and 2023 (163 and 195 individuals, respectively (Fig. 4).

**Fig. 5:**
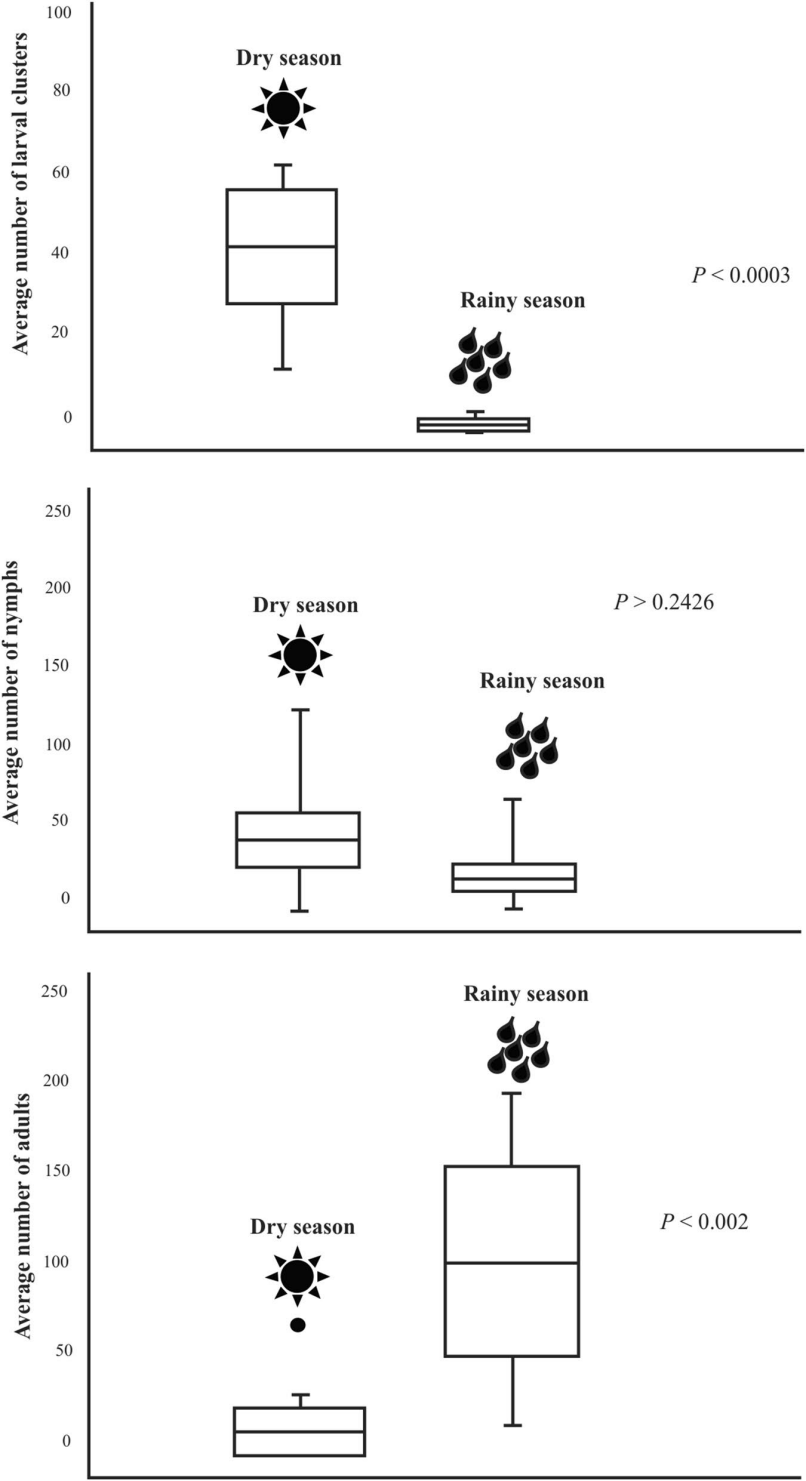
Boxplot of the average number of *Amblyomma cajennense* sensu stricto ticks (larval clusters, nymphs and adults) collected in the dry and rainy seasons in the Amazon biome, municipality of Santa Inês, Maranhão state, Brazil

**Fig. 6:**
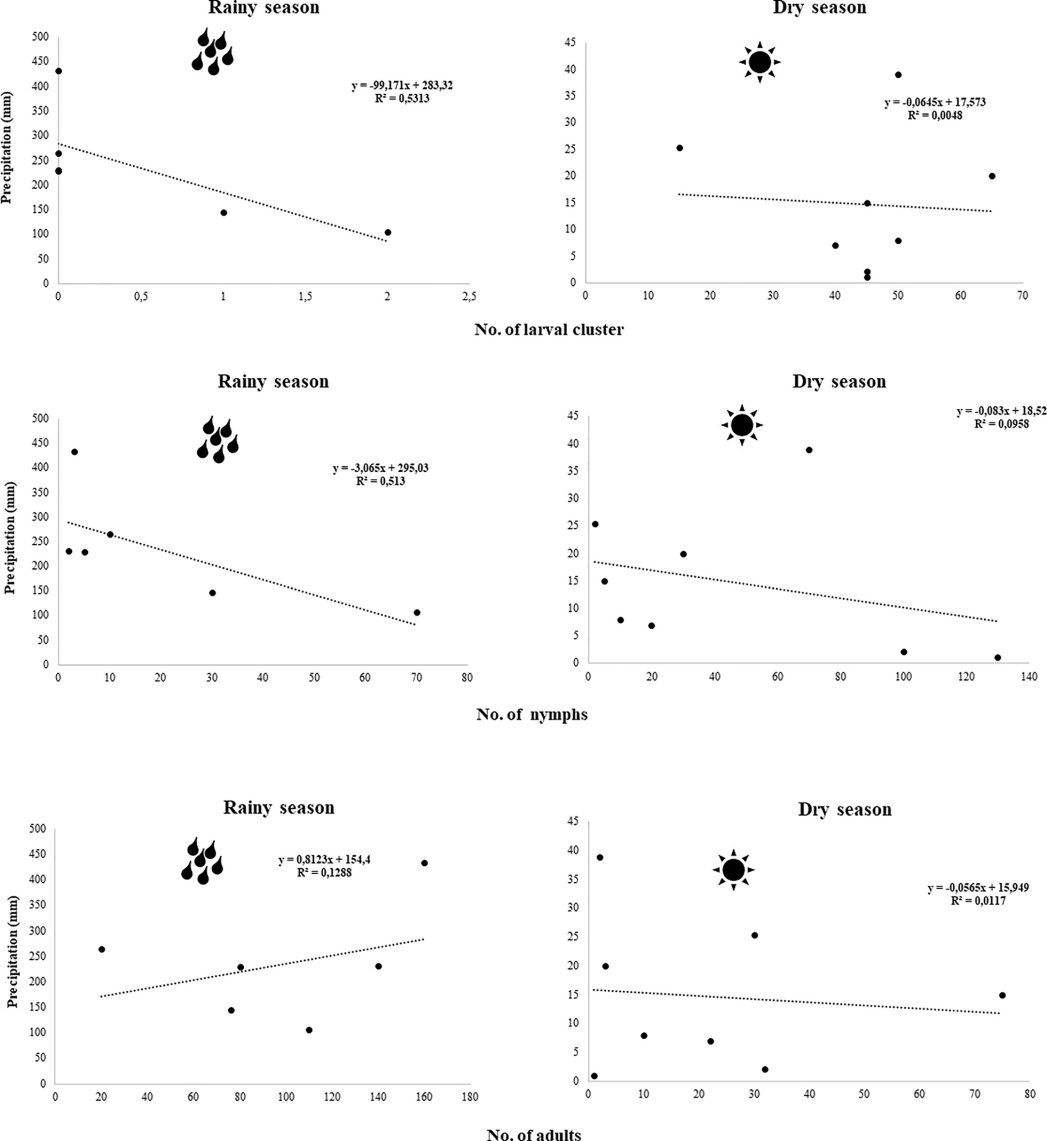
Pearson’s correlation coefficient (*r*) between number of *Amblyomma cajennense* sensu stricto ticks (larval clusters, nymphs and adults) and precipitation (mm), municipality of Santa Inês, Maranhão state, Brazil

Comparisons of the mean number of larval clusters, nymphs and adults in the dry and rainy seasons showed that the larval clusters were significantly more abundant in the dry season than in the rainy season (Kruskal–Wallis H-test, *H* = 36.90,* df* = 1, *P* = 0.0003) (Figs. 5, 6). In contrast, adults were significantly more abundant in the rainy season (Kruskal–Wallis H-test, *H* = 36.90,* df* = 1, *P* = 0.002) (Fig. 5c). Nymphs were more frequent in the transition periods from the dry and rainy seasons, so their abundance was similar in both seasons (Kruskal–Wallis H-test, *H* = 36.90,* df* = 1, *P* = 0.2426) (Figs. 5, 6).

Larval clusters (*n* = 355) were more frequent when the number of daylight hours were shorter (< 12 h) (dry season) (*P* < 0.0002). Although present in all months of the experiment, adults were more frequent under long daylight conditions (> 12 h) (*n* = 1308) (*P* < 0.001). Nymphal abundance was statistically similar among the periods of different daylight hours (*P* = 0.003). In addition, larvae showed a significant negative linear correlation with rainfall (*r* =  − 0.7462; *P* < 0.001; R2 = 0.6417), while adults showed a positive correlation (*r* = 0.8476; *P* < 0.001; R2 = 0.7941). There was no correlation between tick stages and average temperature (°C) and RH (%).

A total of 38.4% (38/99) of adults of *A. cajennense* s.s. tested positive by the real-time PCR assay targeting the rickettsial *gltA* gene. Of these, 33 (14 males, 19 females) were also positive for the conventional PCR targeting the *ompA* gene of SFG-rickettsiae. PCR products of *ompA* gene generated reliable DNA sequences from seven ticks (4 females, 3 males), all of which were 100% identical to sequences of *R. amblyommatis* available in GenBank (accession numbers: MN313363.1, MH818422.1). Based on rickettsial confirmation by DNA sequences, the infection rate by *R. amblyommatis* in *A. cajennense* s.s. ticks was 7.1% (7/99).

Although ticks collected on humans were not quantified, all three active stages (larvae, nymphs and adults) were frequently found on clothing and/or attached to humans in the study area, both on researchers and on farm workers.

## Discussion

The *A. cajennense* species complex has a distribution that ranges from southern USA to northern Argentina [40, 41] and is represented by six nominal species, two of which occur in Brazil: *A. sculptum* (Atlantic Forest, Pantanal, and Cerrado biomes, being rare in the Caatinga) and *A. cajennense* s.s. (restricted to the Amazon biome) [23, 24]. Previous studies have shown that *A. sculptum* is typically found in the Cerrado biome, where the relative humidity is moderate (approx. 65–78% RH) [42]. In contrast, the Amazonian tick *A. cajennense* s.s. prefers higher relative humidity (approx. 90% RH) [42]. Both species occur in Maranhão state—*A. cajennense* s.s. in the Amazon biome and *A. sculptum* in the Cerrado—with their sympatric occurrence being observed in the Amazon-Cerrado transition zone [24, 43]. Although the study area is on the limits of the Amazon-Cerrado transition zone (Fig. 1), *A. cajennense* s.s. was the only species found during the present study, confirming the previous observations of Martins et al. [24]. The absence of *A. sculptum* can be explained by the high relative humidity in the area throughout the year, even in dry periods (approx. 3 months) (Fig. 3). In addition, during the rainy season, the soil becomes predominantly wet, with some areas flooded for hours or days. These characteristics are unfavorable for *A. sculptum*, which prefers areas where the soil is never wet or flooded [44, 45]. Labruna [42] showed experimentally that *A. sculptum* succumbs after being immersed in water, especially for 48–72 h. Therefore, even though our study area is transitional and degraded, it is still not sustainable for *A. sculptum*.

Numerous studies have shown the effect of climate on the bioecology of ticks during the non-parasitic phase, by affecting their survival, inhibiting their host-seeking activity and/or influencing their population density over time and space [6, 14, 16, 46–50]. In this context, climatic factors (e.g. rainfall, temperature and relative humidity) are essential for the survival of *A. cajennense* s.s. in Amazon region. *Amblyomma cajennense* s.s. ticks were collected year-round in the present study; in contrast, the monthly number of larvae, nymphs and adults collected varied. Larval activity was observed from April to December, peaking in the dry season; nymphs predominated from September to November, peaking in the transition period between dry and rainy seasons; and adults peaked in the rainy season. This seasonal pattern is similar to that recorded in the only previous study conducted in the Amazon biome, which was related to *Amblyomma* spp. (including *A. cajennense* s.s.) in the state of Rondônia, western Brazilian Amazon [22]. These results are also similar to those reported for *A. sculptum* in the southeastern and midwestern regions of Brazil (Cerrado and Atlantic Forest biomes), where larvae predominated in the dry season (autumn and winter), nymphs in the dry-rainy transition period (winter-spring) and adults in the rainy season (spring and summer) [51]. Indeed, the results of the present study suggest that *A. cajennense* s.s. completes one generation per year in the eastern Amazonian of Maranhão state, which is similar to the life-cycle recorded for *A. sculptum* in other Brazilian biomes [19].

Larvae of *A. cajennense* s.s. were more abundant during the dry season when the relative humidity was approximately 70% and were absent in the highest rainfall periods. In southeastern Brazil, Labruna et al. [20] reported that the 1-year generation pattern of *A. sculptum* is controlled by the larval behavioral diapause during the spring and summer months (i.e. the rainy season). These authors showed that larvae hatching during the warm and rainy seasons remained inactive and confined under the leaf litter until early autumn (April or May, late rainy season), when they were observed questing on the vegetation. Interestingly, larval activity in our study also started at the end of the rainy season, in May 2022 and April 2023 (i.e. there was no active larvae during the rainy season).

Cabrera and Labruna [21] demonstrated that larval behavioral diapause of *A. sculptum* is primarily controlled by photoperiod. They showed that larvae hatching during the spring and summer (daylight length > 12 h) remains inactive until the next autumn. When either the hours of daylight decrease < 12 h or the mean temperature drops to < 20 °C, larvae terminate the dormancy state and begin their host-seeking activity. In the present study, host-seeking larvae were absent from the vegetation during the period January to March (rainy season) and were first captured during April or May of both 2022 and 2023. This initial larval activity coincided with the hours of daylight decreasing to < 12 h, suggesting that, similarly to *A. sculptum,* the larval behavioral diapause could also be controlling the 1-year generation pattern of *A. cajennense* s.s. Indeed, further studies are needed to confirm such a hypothesis.

Although our primary objective was to understand the seasonal dynamics of *A. cajennense* s.s. in the Amazon biome, 99 adult ticks were also tested for rickettsial agents. In total, 33 (33.3%) ticks tested positive in PCR assays targeting the rickettsial *ompA* gene, which is present only in SFG *Rickettsia* species. DNA sequences were successfully generated for seven out of 33 *ompA*-PCR positive ticks, all of which assigned to *R. amblyommatis*. This bacterium has been detected in 34 tick species in 17 American countries [29]. The infection rate in ticks can exceed 90% in the USA, Panama and Brazil, with approximately 70% of natural infection for the Amazon biome [52]. *Rickettsia amblyommatis* is known to be less prevalent in *A. cajennense* s.s. populations in Maranhão [53], as corroborated by our results. It is important to mention that *R. amblyommatis* is potentially pathogenic to humans [29], but its association with clinical cases of spotted fever rickettsiosis in humans and animals remains unclear. Interestingly, it has been shown that *R. amblyommatis* infection may prevent ticks from acquiring other species of *Rickettsia*, may alter tick host-seeking behavior and may influence the progression of disease symptoms when infecting humans [29]. Altogether, these data emphasize the importance of further studies on this rickettsial organism.

Finally, our study reports for the first time a seasonal behavior of the Amazonian tick *A. cajennense* s.s. The observed pattern of 1-year generation confirms this generation time for *Amblyomma* spp. in the Amazon biome [22]. The finding of *A. cajennense* s.s. adults infected with *R. amblyommatis* suggests that animals and humans are exposed to the risk of infection by this agent, since humans and wild animals are frequent in the study sites. Although the natural hosts of *R. amblyommatis* remain unknown, the presence of infected ticks and capybaras in the present study suggest the possibility that these rodents may be acting as amplifying hosts of this bacterium. Further studies are advocated to better understand the relationship between *A. cajennense* s.s., *R. amblyommatis*, capybaras and humans in the Amazon biome, considering that this ixodid is the primary vector of *R. amblyommatis* and one of the most common ticks parasitizing animals and humans in this region [24, 26, 27].

## Conclusion

Our data suggest a 1-year generation pattern for *A. cajennense* s.s., with a well-defined seasonality of larvae, nymphs and adults in the Amazon biome. Larvae predominate during the dry season, nymphs in the dry/rainy transition and adults in the dry season. The presence of *R. amblyommatis* in adults suggest that animals and humans in the study region are at risk of infection by this SFG-rickettsial agent. Future studies investigating the relationship between *R. amblyommatis*, *A. cajennense* s.s., reservoirs (e.g. capybara) and humans are needed in the Amazon biome.

## Data Availability

Sequences generated in this study are deposited in GenBank under the accession numbers OR289675–OR289681.

## References

[1] Szabó MPJ, Pinter A, Labruna MB (2013). Ecology, biology and distribution of spotted-fever tick vectors in Brazil. Front Cell Infect Microbiol.

[2] Luz HR, Costa FB, Benatti HR, Ramos VN, Serpa A, Martins MC (2019). Epidemiology of capybara-associated Brazilian spotted fever. PLoS Neg Trop Dis.

[3] Bernard Q, Phelan JP, Hu LT. Controlling Lyme disease: new paradigms for targeting the tick pathogen-reservoir axis on the horizon. Front Cell Infect Microbiol. 2020;10:607170. 10.3389/fcimb.2020.607170. 10.3389/fcimb.2020.607170 PMC7744311 33344266

[4] Dias TC, Stabach JA, Huang Q, Labruna MB, Leimgruber P, Ferraz KM (2020). Habitat selection in natural and human-modified landscapes by capybaras (*Hydrochoerus hydrochaeris*), an important host for *Amblyomma sculptum* ticks. PLoS ONE.

[5] Rand PW, Lubelczyl C, Lavigne GR, Elias S (2003). Deer density and the abundance of *Ixodes scapularis* (Acari: Ixodidae). J Med Entomol.

[6] Randolph SE (2004). Tick ecology: processes and patterns behind the epidemiological risk posed by ixodid ticks as vectors. Parasitology.

[7] Boyer PH, Barthel C, Mohseni-Zadeh M, Talagrand-Reboul E, Frickert M, Jaulhac B (2022). Impact of different anthropogenic environments on ticks and tick-associated pathogens in Alsace, a French region highly endemic for tick-borne diseases. Microorganisms.

[8] Randolph SE, Rogers DJ. Towards new population models as tools for the control of African ticks and tick-borne diseases. In: Coons LB, editor. Proceedings of the 2nd international conference on tick-borne pathogens at the host-vector interface. Kruger National Park, South Africa; 1997. pp. 20–35.

[9] Eisen RJ, Eisen L (2018). The blacklegged tick, *Ixodes scapularis*: an increasing public health concern. Trends Parasitol.

[10] Burtis JC, Foster E, Schwartz AM, Kugeler KJ, Maes SE, Fleshman AC (2022). Predicting distributions of blacklegged ticks (*Ixodes scapularis*), lyme disease spirochetes (*Borrelia burgdorferi* sensu stricto) and human lyme disease cases in the eastern United States. Ticks Tick Borne Dis.

[11] Ostfeld RS, Felicia K (2023). Does experimental reduction of blacklegged tick (*Ixodes scapularis*) abundance reduce Lyme disease incidence?. Pathogens.

[12] Sonenshine DE (1993). Biology of ticks.

[13] Sonenshine DE, Atwood EL, Lamb JTJR (1996). The ecology of ticks transmitting Rocky Mountain spotted fever in a study area in Virginia. Ann Entomol Soc Am.

[14] Ogden NH, Lindsay LR (2016). Effects of climate and climate change on vectors and vector-borne diseases: Ticks are different. Trends Parasitol.

[15] Nielebeck C, Kim SH, Pepe A, Himes L, Miller Z, Zummo S (2023). Climatic stress decreases tick survival but increases rate of host-seeking behavior. Ecosphere.

[16] Ogden NH, Lindsay LR, Beauchamp G, Charron D, Maarouf A, O’Callaghan CJ (2004). Investigation of relationships between temperature and developmental rates of tick *Ixodes scapularis* (Acari: Ixodidae) in the laboratory and field. J Med Entomol.

[17] MacDonald AJ (2018). Abiotic and habitat drivers of tick vector abundance, diversity, phenology and human encounter risk in southern California. PLoS ONE.

[18] Reynolds C, Kontschán J, Takács N, Solymosi N, Sándor AD, Keve G (2022). Shift in the seasonality of ixodid ticks after a warm winter in an urban habitat with notes on morphotypes of *Ixodes ricinus* and data in support of cryptic species within *Ixodes frontalis*. Exp Appl Acarol.

[19] Paula LGF, de Nascimento RM, Franco AO, Szabó MPJ, Labruna MB, Monteiro C (2022). Seasonal dynamics of *Amblyomma sculptum*: a review. Parasit Vectors..

[20] Labruna MB, Amaku M, Metzner JA, Pinter A, Ferreira F (2003). Larval behavioral diapause regulates life cycle of *Amblyomma cajennense* (Acari: Ixodidae) in Southeast Brazil. J Med Entomol.

[21] Cabrera RR, Labruna MB (2009). Influence of photoperiod and temperature on the larval behavioral diapause of *Amblyomma cajennense* (Acari: Ixodidae). J Med Entomol.

[22] Labruna MB, Terassini FA, Camargo LMA (2009). Notes on population dynamics of *Amblyomma* ticks (Acari: Ixodidae) in Brazil. J Parasitol.

[23] Nava S, Beati L, Labruna MB, Cáceres AG, Mangold AJ, Guglielmone AA (2014). Reassessment of the taxonomic status of *Amblyomma*
*cajennense* (Fabricius, 1787) with the description of three new species,* Amblyomma tonelliae* n. sp., *Amblyomma*
*interandinum* n. sp. and *Amblyomma*
*patinoi* n. sp., and reinstatement of *Amblyomma** mixtum* Koch, 1844, and *Amblyomma*
*sculptum* Berlese, 1888 (*Ixodida*: *Ixodidae*). Ticks Tick-borne Dis..

[24] Martins TF, Barbieri ARM, Costa FB, Terassini FA, Camargo LMA, Peterka CRL (2016). Geographical distribution of *Amblyomma*
*cajennense* (sensu lato) ticks (*Parasitiformes*: *Ixodidae*) in Brazil, with description of the nymph of A cajennense (sensu stricto). Parasit Vectors..

[25] Gianizella SL, Martins TF, Onofrio VC, Aguiar NO, Gravena W, Nascimento CAR (2018). Ticks (Acari: *Ixodidae*) of the state of Amazonas. Brazil Exp Appl Acarol.

[26] Luz HR, Martins TF, Muñoz-Leal S, Costa FB, Gianizella SL, Faccini JLH, Mikkola H (2020). Ticks from the Brazilian Amazon: species, distribution and host-relations. Ecosystem and biodiversity of Amazonia.

[27] Nogueira BCF, Campos AK, Muñoz-Leal S, Pinter A, Martins TF (2022). Soft and hard ticks (*Parasitiformes*: *Ixodida*) on humans: a review of Brazilian biomes and the impact of environmental change. Acta Trop.

[28] Benatti HR, Binder LC, Costa FB, Soares HS, Luz HR, Labruna MB (2020). Maintenance of the infection by *Rickettsia amblyommatis* in *Amblyomma cajennense* sensu stricto ticks and evaluation of vector competence. Exp App Acarol.

[29] Richardson EA, Roe RM, Apperson CS, Ponnusamy L (2023). *Rickettsia amblyommatis* in ticks: a review of distribution, pathogenicity, and diversity. Microorganisms.

[30] ZEE - Sumário Executivo do Zoneamento Ecológico Econômico do Estado do Maranhão –ZEE: etapa Bioma Amazônico. Paulo Henrique de Aragão Catunda; Luiz Jorge Bezerra da Silva Dias (organizadores). São Luís: IMESC; 2019.

[31] Terassini FA, Barbieri FS, Albuquerque S, Szabó MP, Camargo LM, Labruna MB (2010). Comparison of two methods for collecting free-living ticks in the Amazonian forest. Ticks Tick Borne Dis.

[32] Szabó MP, Labruna MB, Garcia MV, Pinter A, Castagnolli KC, Pacheco RC (2009). Ecological aspects of the free-living ticks (Acari: Ixodidae) on animal trails within Atlantic rainforest in south-eastern Brazil. Ann Trop Med Parasitol.

[33] Ramos VN, Osava CF, Piovezan U, Szabó MPJ (2014). Complementary data on four methods for sampling free-living ticks in the Brazilian Pantanal. Rev Bras Parasitol Vet.

[34] Martins TF, Onofrio VC, Barros-Battesti DM, Labruna MB (2010). Nymphs of the genus *Amblyomma* (Acari: Ixodidae) of Brazil: descriptions, redescriptions, and identification key. Ticks Tick-borne Dis.

[35] Dantas-Torres F, Martins TF, Muñoz-Leal S, Onofrio VC, Barros-Battesti DM (2019). Ticks (*Ixodida*: *Argasidae*, *Ixodidae*) of Brazil: updated species checklist and taxonomic keys. Ticks Tick-borne Dis.

[36] Sangioni LA, Horta MC, Vianna MCB, Gennari SM, Soares RM, Galvão MAM (2005). Rickettsial infection in animals and Brazilian spotted fever endemicity. Emerg Infect Dis.

[37] Soares JF, Soares HS, Barbieri AM, Labruna MB (2012). Experimental infection of the tick *Amblyomma cajennense*, Cayenne tick, with *Rickettsia rickettsii*, the agent of Rocky Mountain spotted sever. Med Vet Entomol.

[38] Regnery RL, Spruill CL, Plikaytis BD (1991). Genotypic identification of rickettsiae and estimation of intraspecies sequence divergence for portions of two rickettsial genes. J Bacteriol.

[39] Mangold AJ, Bargues MD, Mas-Coma S (1998). Mitochondrial 16S rDNA sequences and phylogenetic relationships of species of *Rhipicephalus* and other tick genera among Metastriata (Acari: Ixodidae). Parasitol Res.

[40] Estrada-Peña A, Guglielmone AA, Mangold AJ (2004). The distribution and ecological preferences of the tick *Amblyomma cajennense* (Acari: Ixodidae), an ectoparasite of humans and other mammals in the Americas. Ann Trop Med Parasitol.

[41] Beati L, Nava S, Burkman EJ, Barros-Battesti D, Labruna MB, Guglielmone AA (2013). *Amblyomma cajennense* (Fabricius, 1787) (Acari: Ixodidae), the Cayenne tick: phylogeography and evidence for allopatric speciation. BMC Evol Biol.

[42] Labruna MB (2018). Comparative survival of the engorged stages of *Amblyomma cajennense* sensu stricto and *Amblyomma sculptum* under different laboratory conditions. Ticks Tick Borne Dis.

[43] Costa FB, Martins TF, Muñoz-Leal S, Serpa MCA, Ogrzewalska M, Luz HR (2020). Retrospective and new records of ticks (Acari: Argasidae, Ixodidae) from the state of Maranhão, an Amazon-Cerrado transition area of Brazil. Vet Parasitol Reg Stud Rep.

[44] Szabó MP, Castro MB, Ramos HG, Garcia MV, Castagnolli KC, Pinter A (2007). Species diversity and seasonality of free-living ticks (Acari: ixodidae) in the natural habitat of wild Marsh deer (*Blastocerus dichotomus*) in Southeastern Brazil. Vet Parasitol.

[45] Queirogas VL, Del Claro K, Nascimento AR, Szabó MP (2012). Capybaras and ticks in the urban areas of Uberlândia, Minas Gerais, Brazil: ecological aspects for the epidemiology of tick-borne diseases. Exp Appl Acarol.

[46] Campbell A, Glines MV (1979). Development, survival, and oviposition of the rabbit tick, *Haemaphysalis leporispalustris* (Packard) (Acari: *Ixodidae*), at constant temperatures. J Parasitol.

[47] Peavey CA, Lane RS (1996). Field and laboratory studies on the timing of oviposition and hatching of the western black-legged tick, *Ixodes pacificus* (Acari: Ixodidae). Exp Appl Acarol.

[48] Chilton NB, Andrews RH, Bull CM (2000). Influence of temperature and relative humidity on the moulting success of *Amblyomma limbatum* and *Aponomma hydrosauri* (Acari: *Ixodidae*) larvae and nymphs. Int J Parasitol.

[49] Eisen L, Eisen J, Lane RS (2002). Seasonal activity patterns of *Ixodes pacificus* nymphs in relation to climatic conditions. Med Vet Entomol.

[50] Herrmann C, Gern L (2010). Survival of *Ixodes ricinus* (Acari: Ixodidae) under challenging conditions of temperature and humidity is influenced by *Borrelia burgdorferi* sensu lato infection. J Med Entomol.

[51] De Paula LGF, Sampaio ALN, Zeringota V, Bezerra G, Barreto ALG, Santos AA (2021). Seasonal dynamics of *Amblyomma sculptum* (Acari: *Ixodidae*) in the Cerrado biome of midwestern Brazil. Exp Appl Acarol.

[52] Soares HS, Barbieri ARM, Martins TF, Minervino AHH, de Lima JTR, Marcili A (2015). Ticks and rickettsial infection in the wildlife of two regions of the Brazilian Amazon. Exp Appl Acarol.

[53] Costa FB, Costa AP, Moraes-Filho J, Martins TF, Soares HS, Ramirez DG (2017). *Rickettsia*
*amblyommatis* infecting ticks and exposure of domestic dogs to *Rickettsia* spp in an amazon-cerrado transition region of northeastern Brazil. PLoS One..

[54] Marques EQ, Marimon-Junior BH, Marimon BS, Matricardi EAT, Mews HA, Colli GR (2020). Redefning the cerrado-amazonia transition: implications for conservation. Biodivers Conserv.

